# Correction: NanoSIMS single cell analyses reveal the contrasting nitrogen sources for small phytoplankton

**DOI:** 10.1038/s41396-020-00813-w

**Published:** 2020-10-23

**Authors:** Hugo Berthelot, Solange Duhamel, Stéphane L’Helguen, Jean-Francois Maguer, Seaver Wang, Ivona Cetinić, Nicolas Cassar

**Affiliations:** 1 Laboratoire des Sciences de l’Environnement Marin (LEMAR), UMR 6539 UBO/CNRS/IRD/IFREMER, Institut Universitaire Européen de la Mer (IUEM), Brest, France; 2 Division of Biology and Paleo Environment, Lamont-Doherty Earth Observatory, PO Box 1000, 61 Route 9W, Palisades, NY 10964 USA; 3 Division of Earth and Ocean Sciences, Nicholas School of the Environment, Duke University, Durham, NC 27708 USA; 4 NASA Goddard Space Flight Center, Ocean Ecology Laboratory, Code 616, Greenbelt, MD USA; 5 GESTAR/Universities Space Research Association, Columbia, MD USA

**Keywords:** Biogeochemistry, Microbial biooceanography, Microbial ecology

Correction to: *The ISME Journal*

10.1038/s41396-018-0285-8

This article was originally published under NPG’s License to Publish, but has now been made available under a CCBY license. The PDF and HTML versions of the paper have been modified accordingly.

